# Supplementary material for: Patterns of Intron Gain and Loss in Fungi
Source: PLoS Biol. 2004 Nov 30;2(12):e422. doi: 10.1371/journal.pbio.0020422 (PMC532390; doi:10.1371/journal.pbio.0020422)
Supplement: Table S1 — Also available at http://genes.mit.edu/NielsenEtAl/. (4.3 MB ZIP). [file pbio.0020422.st001.zip › NielsenEtAl/html/1306.html]

AN5732.1.NCU05421.1.MG03282.1.FG01642.1


```
 CLUSTAL W (1.82) Multiple Sequence Alignments - Introns Inserted


Sequence 1: AN5732.1	437 aa
Sequence 2: FG01642.1	399 aa
Sequence 3: NCU05421.1	433 aa
Sequence 4: MG03282.1	429 aa
Alignment Length: 488 aa
Number Identitical Residues: 121 aa
Alignment Score (without introns) 5676


MG03282.1 	MATAATR-----IRTLGPG-----------SSLAKEAMRALRGENLTRHAMDERNK-LQI
NCU05421.1	MAAVLLRNTTCGIRCLTKGPAFISTTATISSSHGFGLLTKLRRSYGTTISQDLLNKKVQV
FG01642.1 	MMLLKRPLQNSTPRSIPR---------------LIPSLSTIPRRQFTDAASHSSNK-IQV
AN5732.1  	MVLFSQATCWKAYRFKKSR-----------GGLFVRFNSAFSSDRFAQLASRPASI-HQI
          	*            *                ..        :     :  :    .   *:

MG03282.1 	YLSTEKDPYLNLAAEQHLLEVSHPSSTVLFMYINRPSVIIGRSQNPWLEVNLGRLGSGLP
NCU05421.1	YQSTSKDPYLNLSIEHHLLQHSHPESYVLFLYINDPCVVIGRNQNPWLEVNL-------P
FG01642.1 	YTSKSRDPFLNLSVEHYLLQKTPPESTILFLYTNDPCIVFGRNQNPWMEVNL-------P
AN5732.1  	YQSLSTDPYVNLSIEHFLLEKAPPDSSILFLYVNRPCVVIGRNQNPWLETNL-------Q
          	* * . **::**: *:.**: : *.* :**:* * *.:::**.****:*.**        

MG03282.1 	RLSAKQDE--EEAQQHVPVSLVRRRSGGGTVFHDHGNVNWSVIFPTAEFDRDRHAEMVVR
NCU05421.1	ALQEAED-----------IKLVRRRSGGGTVFHDHGNVNWSVICPPAVFDRDRHAEMVVR
FG01642.1 	RLDKFRNDPASVGWTGGPVQLVRRRSGGGAVFHDEGNVNFSVICPPAVFDRNKHAEMVVR
AN5732.1  	KLHNDREE--STKHSDG-ALLVRRRSGGGAVFHDAGNLNYSVISPRATFTRNKHAEMMMR
          	 *   .:.  .         *********:**** **:*:*** * * * *::****::*

MG03282.1 	ALRDRIGVPTVRVNDRHDIVMDVPRKPFSEENKTFKVSGSAYRVTRVRSLHHGTCLVHSP
NCU05421.1	ALKD-LGVTTAKVNERHDIVIAGDGRGNGQD--IFKVSGSAYKLTRLRSLHHGTCLLNSP
FG01642.1 	ALSS-LGKPNTRVNERHDIVMDIPNDP----IGTYKISGSAYKLTRLRSLHHGTCLLRSP
AN5732.1  	ALHR-VGAVNTSVNERHDIVMSESDG------QPRKISGSAFKLTRFRGLHHGTCLLDSP
          	**   :*  .. **:*****:              *:****:::**.*.*******: **

MG03282.1 	YVDQIGPLLRPPMAGSYLKTRGAGSVRSPVRNVSGGTQFDEVAFGTPFFQAMVIDEFKET
NCU05421.1	RLKNIGKFLRSPGE-PYIKARGVESVRSPIRNVD---------VDTEEFTKGVVDEFRAM
FG01642.1 	NLKNISGMLRSPAE-PFIKTRGVDSVRSPVRNVG--IENA-------AFEGAVLEEFARM
AN5732.1  	NINELGSFLRSPAR-EYIRAKGVESVRSPVANVSSSMEDAPAGFSMQAVIASVMDEFAQL
          	 :.::. :**.*    :::::*. *****: **... .    . .   .   *::**   

MG03282.1 	YS--HLSGPGNELVYSIG----------------PSVMTIPDVEKGYKELT0SAAWVYNQ
NCU05421.1	YE--EQCG-EDVTVKCVGE---------------EDVKNMDNIRKGMDELT0SIPWIYGQ
FG01642.1 	YG---DAQVREEISDKVR--------------------GLDVIRKGYDELQ~SRDWIYGQ
AN5732.1  	YNASPDAVRRAQRAHAVEPELYAGDNWVAGAVGDLEADAVPEIKKGMDELK0SLEWKYTQ
          	* ::  .         :  .  :...  :.: .. .   :  :.** .**  *  * * *

MG03282.1 	TPQFTFSTHPTEMDPRKRPPPPSGVLPT0FQANMTIRYGKITECSFKGLVYD--REDTAV
NCU05421.1	TPQFTFSSRPTEDDPRERPPLDLDVQSD0FNVQFTLRHGEIQTANVSGLKYEGTAMDGDI
FG01642.1 	TPRFTFSSYPYAEDPRER----PQLDFD0TKLRFDARHGVVEK-------FTAQGASSAT
AN5732.1  	TPQFTFSTYPIEEDPRERPALPPSLPPS0TRVFLRLKHGAIIES----CISTSNDPSLAA
          	**:****: *   ***:*..    :     .  :  ::* :  .        .   .   

MG03282.1 	NEGLR------GRE------LHSIT--DWRQTLAELSPAQVPN------NATGDWLNKVF
NCU05421.1	YDGMISQTLAKGSEDDSAVKLYEIK--DWATVLKSATAGQAFKVSDSSLNSISEWLNKVF
FG01642.1 	EQGD--LSALASSS------IYDV--SSWTAQLSQAGISDGEA------VKFGSWMDNVL
AN5732.1  	EQASRVHEALKGRN------LHELQPSQWTEVLVSRLSADEEP------VTVQELAS-FI
          	 :.        . .      ::.: .:.*   * .   .:             .  . .:

MG03282.1 	GNTPEEKTSS
NCU05421.1	PGNHFR----
FG01642.1 	GVDFTKPTQ-
AN5732.1  	TSKFGS----
          	
```
